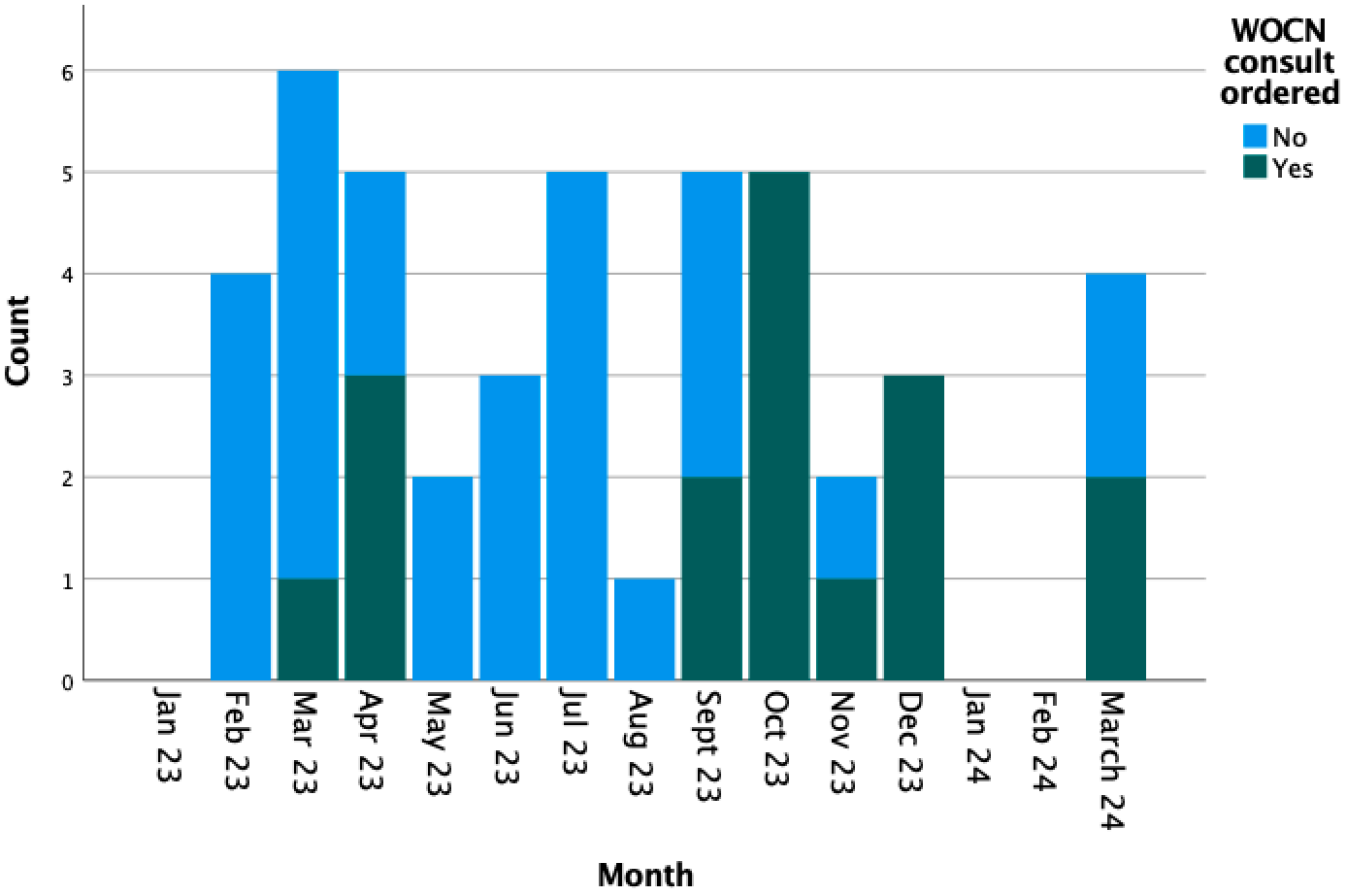# 12 Decreasing the Pressure: Multi-pronged Nursing-Driven Protocol for Hospital Acquired Pressure Injury Prevention in Burn Patients

**DOI:** 10.1093/jbcr/iraf019.012

**Published:** 2025-04-01

**Authors:** Julie Childers, Carey Lamphier, Alicia Smith, Victoria Sheesley, Yuk Ming Liu, Laura Johnson, Lauren Nosanov

**Affiliations:** Grady Hospital; Grady Hospital; Grady Health System; Grady Hospital; Emory University - Walter L. Ingram Burn Center; Walter L. Ingram Burn Center at Grady Memorial Hospital; Emory University School of Medicine

## Abstract

**Introduction:**

Healthcare-acquired pressure injuries (HAPI) present a significant challenge in the care of burn patients, adding complexity and increasing morbidity. Within the setting of already inherently compromised skin integrity, factors such as prolonged immobility, critical illness, and utilization of medical devices have been shown to increase the incidence of HAPI. Nursing-driven protocols for patient repositioning and invasive device monitoring as well as attentiveness to thorough documentation may help mitigate risk. This quality improvement study investigates the impact of initiation of new nursing-driven protocols on the prevalence, characteristics, and clinical outcomes of HAPI in patients admitted to a high-volume regional Burn Center.

**Methods:**

Concern for elevated HAPI rates, underutilization of Wound, Ostomy, and Continence Nursing (WOCN) consultation services, and inconsistent documentation spurred the introduction in September, 2023 of the Turn Your Burn program as well as in-service training on Heel Medics boots. Patients with HAPI were identified from the Burn Center registry for 8 months pre-implementation and 6 months post-implementation. Retrospective chart review collected data on patient demographics, burn injury characteristics, patient acuity, HAPI characteristics and timing of diagnosis, utilization of WOCN consultation, and associated clinical outcomes. Descriptive statistics were reported, and univariate analysis compared pre- and post-implementation groups.

**Results:**

Throughout the 14-month study period (1/2023-3/2024), 45 HAPI were identified in 23 patients admitted to the Burn Unit. Patients were predominantly male (78.3%) with a mean age of 54.4 ± 20.1 years. Most were critically ill at the time of HAPI identification (78.3%), with mean Total Body Surface Area % (TBSA) 34.7% ± 24.1%. Heels and other locations on the feet accounted for 55.6% of cases, while the sacrum was involved in 13.3%. Medical devices were implicated in 26.7%. No HAPI was present at the time of admission, and the median time of diagnosis was hospital day 22.0 (IQR 38.0). Surgical intervention was required in 8.9%. Rates of WOCN involvement significantly increased following protocol implementation (68.4% vs. 15.4%, p < 0.001). Documentation of staging as well as addition of the injury to the Lines-Drains-Airways avatar (LDA) has been steadily increasing over time.

**Conclusions:**

HAPI remains prevalent in burn patients. Implementing targeted interventions, such as Turn Your Burn, in collaboration with Wound, Ostomy, and Continence Nurses (WOCN), can help mitigate HAPI risk in this vulnerable patient population through increased awareness and improved documentation.

**Applicability of Research to Practice:**

Nursing-driven quality improvement efforts through protocol development, implementation, and optimization allow for improved patient care as well as staff engagement.

**Funding for the Study:**

N/A